# Difference in Visual Processing Assessed by Eye Vergence Movements

**DOI:** 10.1371/journal.pone.0072041

**Published:** 2013-09-19

**Authors:** Maria Solé Puig, Laura Puigcerver, J. Antonio Aznar-Casanova, Hans Supèr

**Affiliations:** 1 Department of Basic Psychology, Faculty of Psychology, University of Barcelona, Barcelona, Spain; 2 Institute for Brain, Cognition and Behavior, University of Barcelona, Barcelona, Spain; 3 Catalan Institution for Research and Advanced Studies, Barcelona, Spain; University of Lincoln, United Kingdom

## Abstract

Orienting visual attention is closely linked to the oculomotor system. For example, a shift of attention is usually followed by a saccadic eye movement and can be revealed by micro saccades. Recently we reported a novel role of another type of eye movement, namely eye vergence, in orienting visual attention. Shifts in visuospatial attention are characterized by the response modulation to a selected target. However, unlike (micro-) saccades, eye vergence movements do not carry spatial information (except for depth) and are thus not specific to a particular visual location. To further understand the role of eye vergence in visual attention, we tested subjects with different perceptual styles. Perceptual style refers to the characteristic way individuals perceive environmental stimuli, and is characterized by a spatial difference (local vs. global) in perceptual processing. We tested field independent (local; FI) and field dependent (global; FD) observers in a cue/no-cue task and a matching task. We found that FI observers responded faster and had stronger modulation in eye vergence in both tasks than FD subjects. The results may suggest that eye vergence modulation may relate to the trade-off between the size of spatial region covered by attention and the processing efficiency of sensory information. Alternatively, vergence modulation may have a role in the switch in cortical state to prepare the visual system for new incoming sensory information. In conclusion, vergence eye movements may be added to the growing list of functions of fixational eye movements in visual perception. However, further studies are needed to elucidate its role.

## Introduction

Attention is a neural mechanism for selecting relevant sensory information for further visual information processing. The neural circuits of attention are closely linked to the oculomotor system, e.g. [1]. A shift of attention is usually followed by a saccadic eye movement to shift the eye gaze towards the attended location. Visual attention also relates to small fixational saccades that do not change the focus of eye gaze, where the direction of micro-saccades may uncover the orientation of covert visual attention [2,3]. The function of fixational eye movements is not limited to attention but also includes the prevention of the loss of conscious vision [4], the improvement of visual acuity [5,6], the reduction of binocular disparity [7], and the adjustment of eye positions after a target saccade [8].

**Figure 1:**
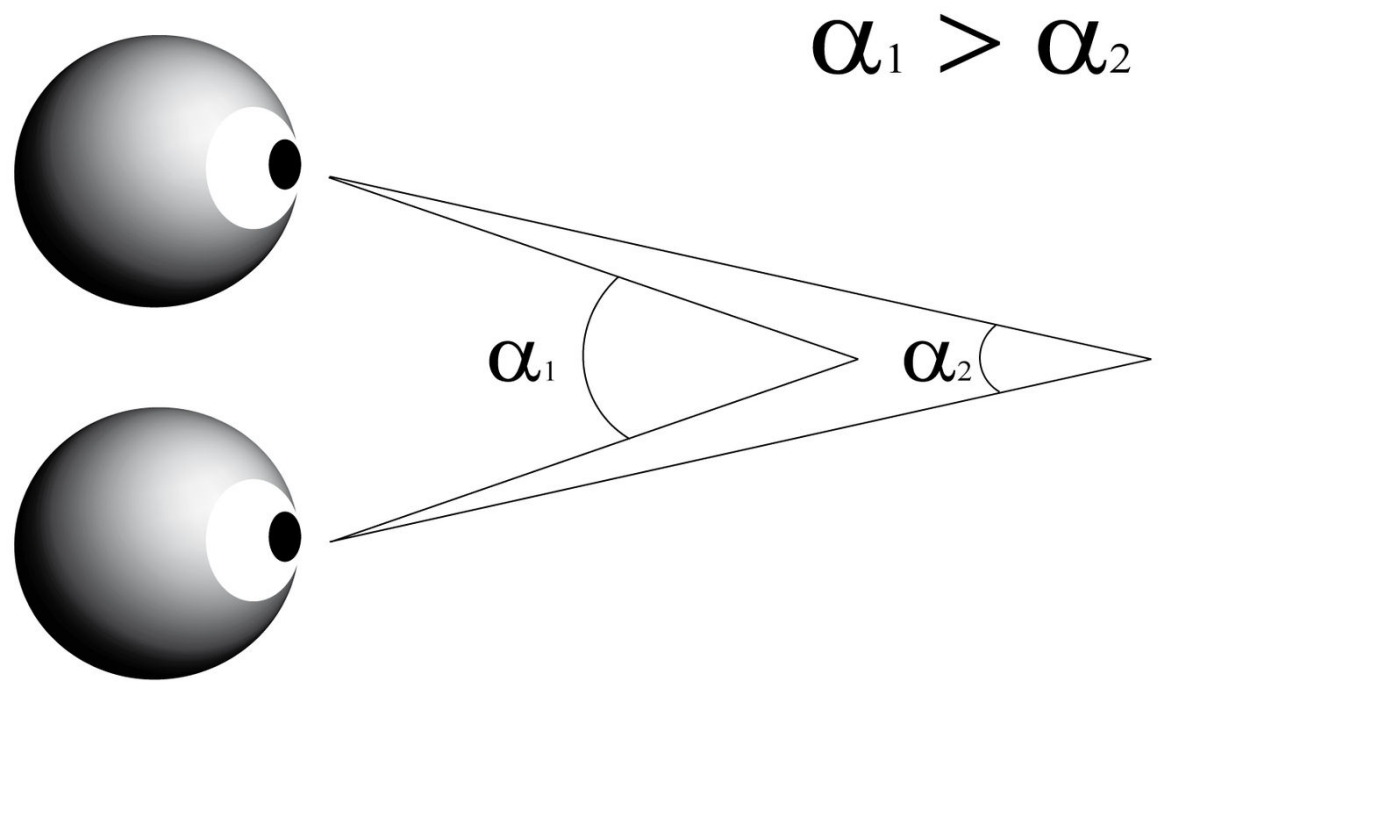
Schematic explanation of the angle of eye vergence. The eyes focus on a single point in space. The angle of eye vergence relates to the distance of the focus point to the eyes. For a near point the vergence angle (α1) is larger than for a far point (α2). α represents the angle of eye vergence.

Recently we found an unpredicted but clear relation of another type of eye movement namely eye vergence movements with visual attention [9]. Vergence refers to the simultaneous movement of both eyes in opposite directions to obtain single binocular vision. When the eyes rotate towards each other (convergence) the angle of eye increases and when the eyes rotate away from each other (divergence) the angle becomes smaller (Figure 1). We observed that during steady gaze fixation visual stimuli modulate the angle of eye vergence, and when orienting attention the eyes briefly converge to a nearer plane, i.e. the vergence angle increases after visual stimulation. This modulation in eye vergence is not a near triad-effect, neither related to pupil size, and is independent of the occurrence of micro-saccades [9]. Instead the increase in vergence angle correlates with bottom-up and top-down induced shifts in visuospatial attention [9]. For instance, vergence angle strongly modulates after cueing the target location but not for uncued targets. Unlike micro- saccades, however, eye vergence movements do not carry directional information of the target because of the nature of such movements.

Therefore, to further understand the relation between vergence eye movements and spatial attention, we made use of people with different perceptual styles. Perceptual style refers to the characteristic way individuals perceive environmental stimuli, and is characterized by the differentiation according to local and global processing [10-13]. Individuals with a global perceptual style have problems to ignore the spatial context of a stimulus (field dependent observers; FD) while individuals with a local style are not so influenced by surrounding stimuli (field independent observers; FI). Perceptual style is attributed to differentiation in brain organization [14] that may result in distinctive attention and spatial abilities [15-18].

We found that eye vergence movements differ among people with different perceptual styles. As perceptual is style is mainly characterized in terms of visuo-spatial processing, this finding may suggest that vergence is relevant to spatial attention [19,20]. Alternatively modulation in vergence eye movements may have a role in the switch in cortical state [21].

## Results

### Behavioral performance

**Figure 2:**
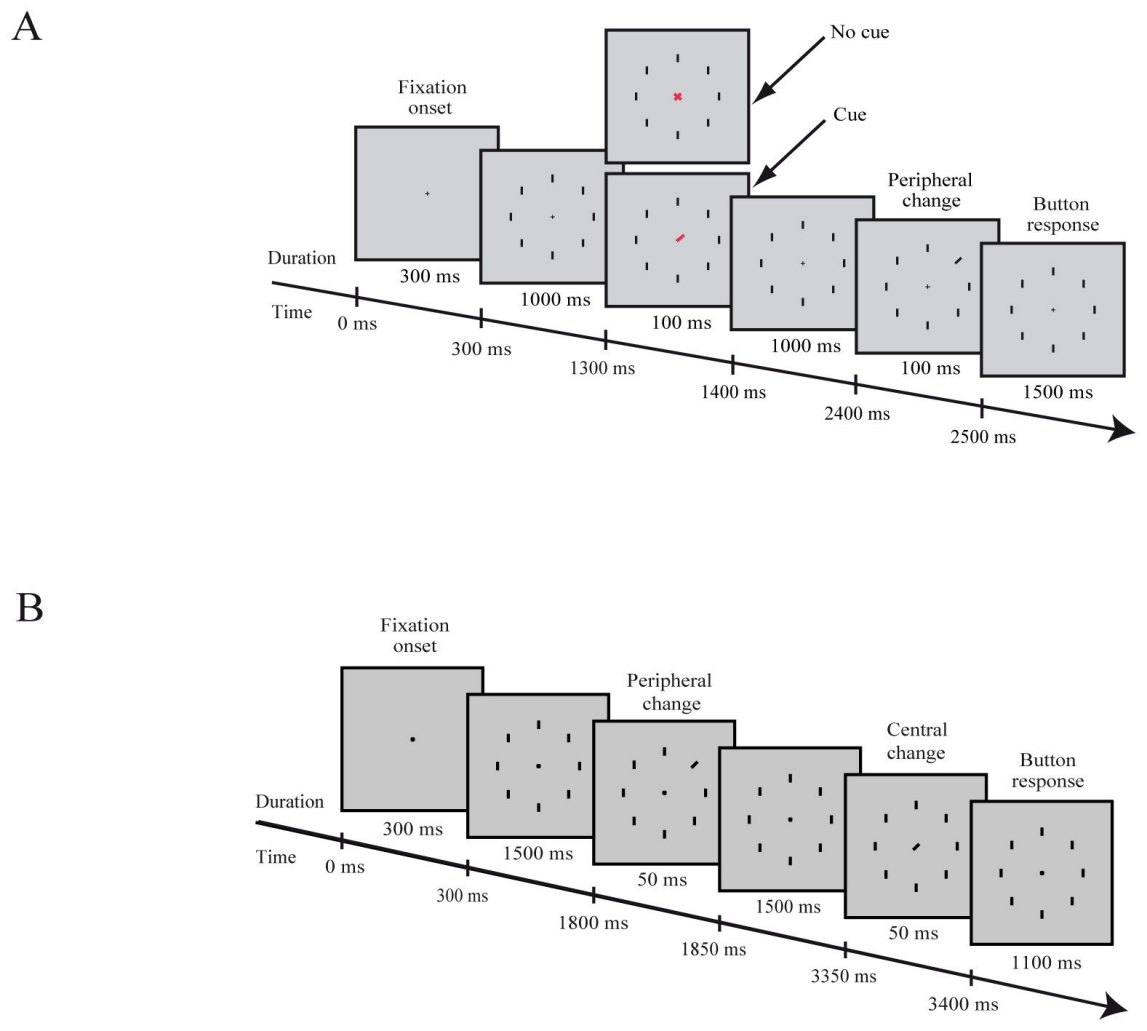
Illustration of the tasks. **A**: The cue/no-cue task. **B** The matching task. Time is from fixation onset.

**Figure 3:**
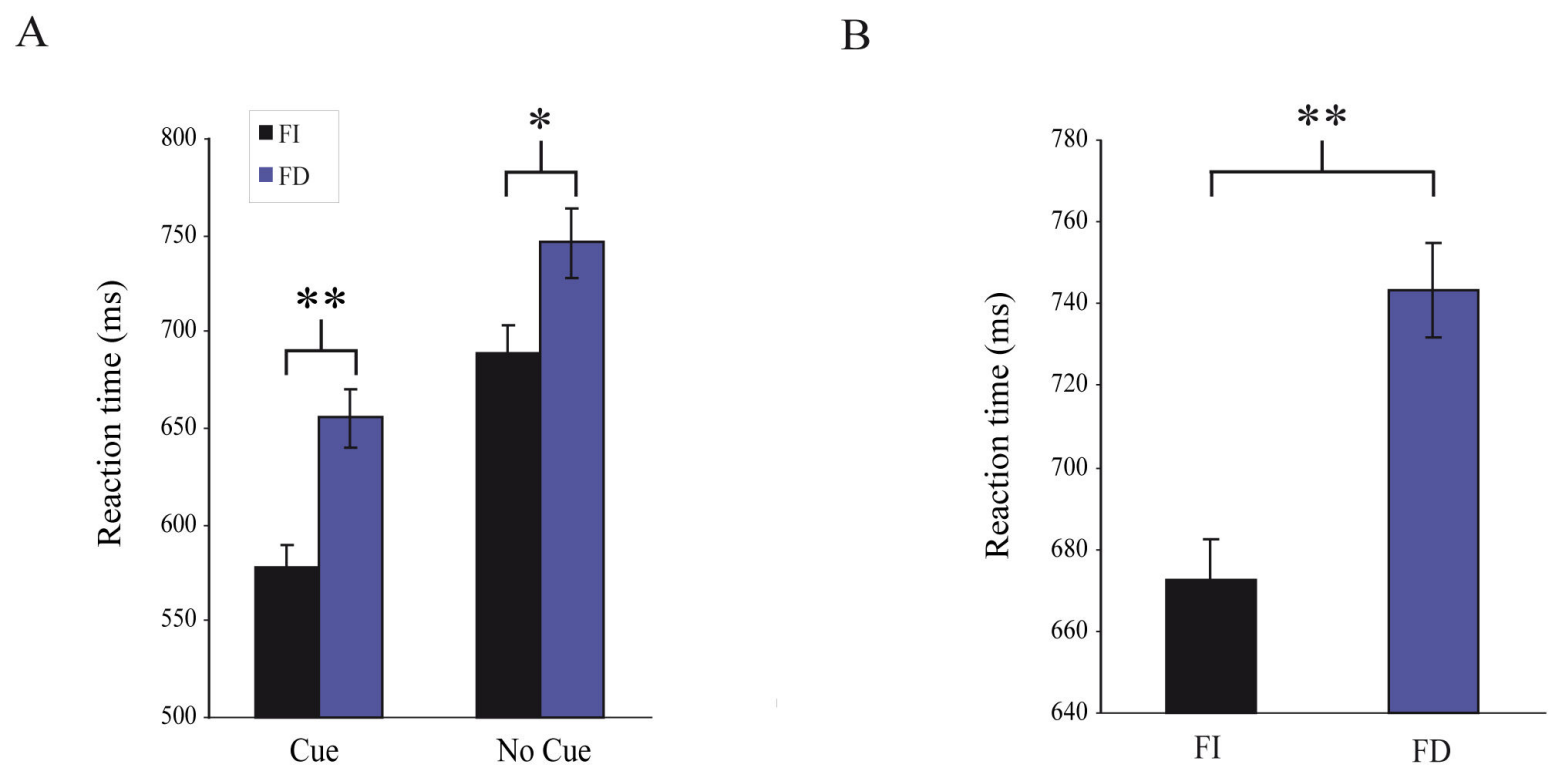
Behavioral performance. Average reaction times from the cue/no-cue task (**A**) and from the matching task (**B**). Error bars are SEM.

In task 1 (Figure 2a), the average response time was significantly shorter in FI group compared to FD group (mean±sem: 633.2±9.8 ms vs. 696.8±11.9 ms; t-test _(687)_ =-4.13, p<0.001). We found that observers from both groups responded faster in the cue condition than in the no-cue condition (mean±sem; FI: 578±11 ms vs. 689±14 ms; FD: 655±15 ms vs. 746±18 ms; t-test _(714)_ =-6.5; p<0.001; Figure 3a). Thus on average FI subjects are 78 ms and 57 ms faster in the cue and no-cue condition, respectively. Regarding the detection performance, FI and FD observers had similar (X^2^-test _(1073)_ =2.92; p=0.09) percentage of correct responses (correct responses; FI: 86.1%; FD: 81.6%).

In task 2 (Figure 2b), we found that FI observers responded significantly faster (71 ms) compared to FD observers (mean±sem: 743±12 ms vs. 672±10 ms; t-test _(481)_ =-4.62; p<0.001; Figure 3b). Percentage of correct responses was higher for FI observers (72.7%) compared to the FD observers (54.8%) X^2^-test _(746)_ =25.62; p<0.001)).

### Eye vergence

**Figure 4:**
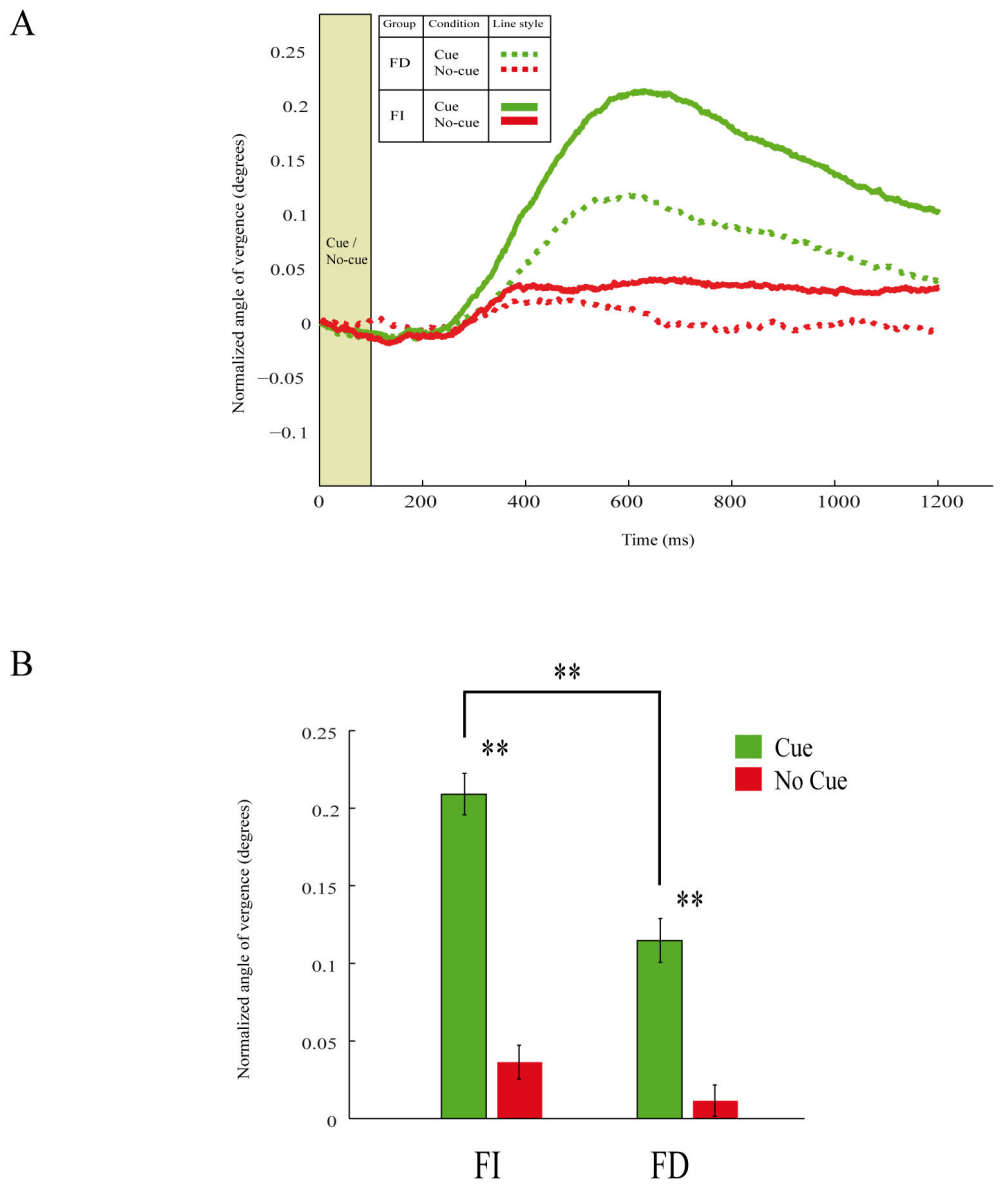
Modulation in eye vergence while performing the cue/no-cue task. **A**: Average modulation in the angle of eye vergence from all subjects in the cue (green) and no-cue (red) conditions for FI (continuous lines) and FD (dotted lines) subjects. Higher values of vergence angle represent convergence. Time is from cue/no-cue onset. **B**: Mean modulation in eye vergence for FI and FD subjects. Asterisks denote significant (p<0.01) differences. Error bars are SEM.

**Figure 5:**
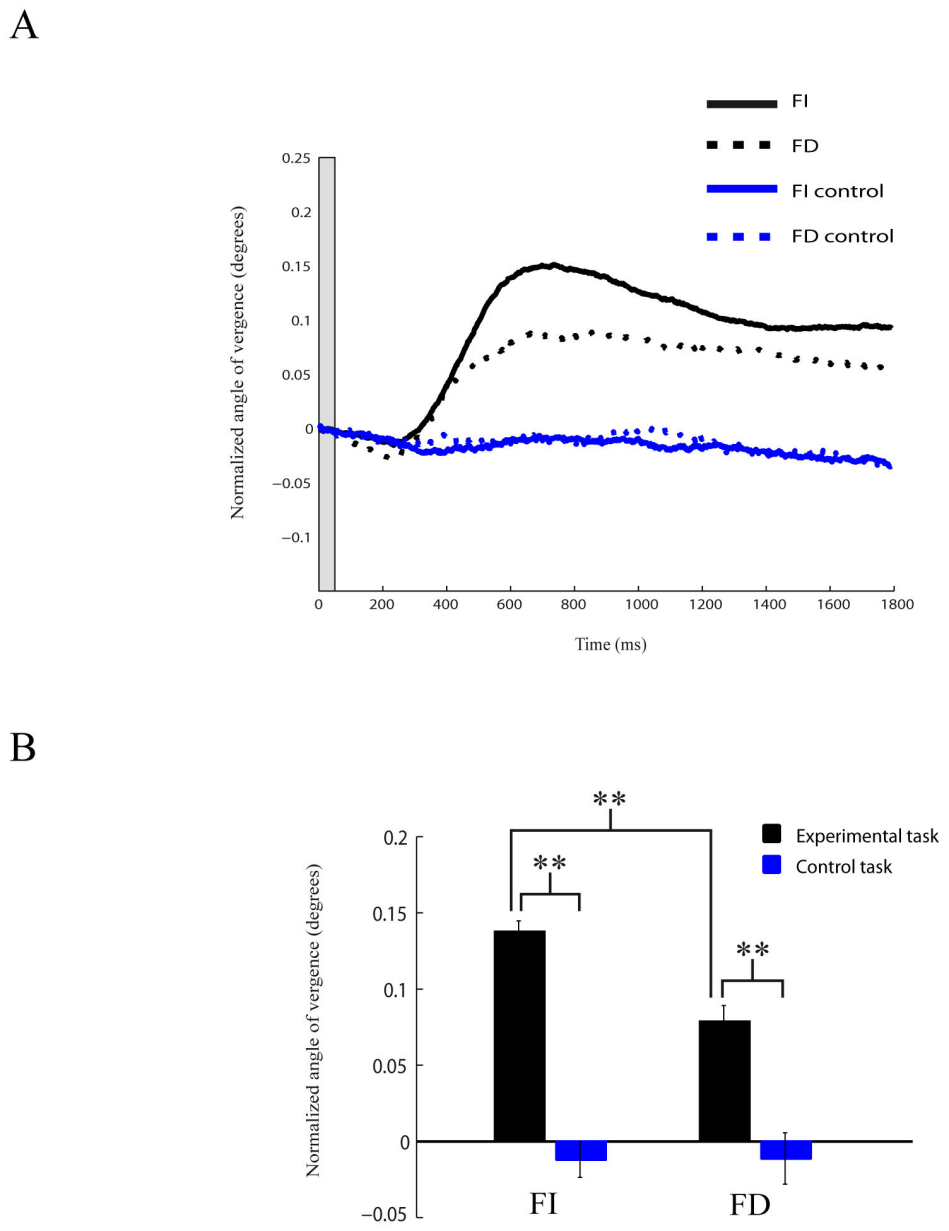
Modulation in eye vergence while performing the matching task. **A**: Average modulation across all subjects in the angle of eye vergence during the task (black) and control task (blue) for FI (continuous lines) and FD (dotted lines) subjects. Higher values of vergence angle represent convergence. Time is from the onset of the peripheral change **B**: Mean modulation in eye vergence for FI and FD subjects. Asterisks denote significant (p<0.01) differences. Error bars are SEM.

The positions of both eyes were simultaneously monitored to compute the angle of eye vergence during fixation. As previously described, we found in the first task that the angle of eye vergence was higher in the cue condition compared to no-cue condition for FI and FD subjects (FI: t-test _(356)_=10.03, p<0.01; FD: t-test _(356)_=5.76, p<0.01; Figure 4). Thus the eyes start to converge after the presentation of the cue stimulus. We compared the strength in the modulation of the angle of eye vergence between FI and FD subjects. For the cue condition, the angle of eye vergence was larger in FI subjects compared to the vergence angle in FD subjects (t-test _(372)_=4.84, p<0.01; Figure 4). No differences in vergence modulation between groups were found in no-cue condition (t-test _(340)_=1.67, p>0.05; Figure 4). We next analyzed the modulation in the angle of eye vergence during the matching task. Also here the modulation in the angle of eye vergence was stronger in FI subjects than in FD subjects (t-test _(480)_=4.82, p<0.01; Figure 5). Thus FI subject show stronger eye convergence than FD subjects. When FI and FD subjects viewed the same visual stimulation sequence but without any instructed task (control task), no difference in vergence modulation between FI and FD subjects was observed (t-test _(200)_=-0.0361, p>0.05; Figure 5).

## Discussion

To better understand the role of vergence eye movements in visuospatial attention, we tested people with different perceptual styles (FI and FD observers) in two paradigms that involve orienting attention, and analyzed the modulation in the angle of eye vergence. In both tasks we observed that FI observers responded faster and were more accurate than FD subjects. We also found that both FI and FD subject had stronger modulation in eye vergence in the cue condition than in the no-cue condition. Furthermore, we observed that in the cue condition of the first task and in the experimental condition of the second task, FI subjects have a stronger modulation in the angle of eye vergence than FD subjects. Thus observers converge (and not diverge) their eyes after cue/stimulus presentation where FI subjects show stronger convergence than FD subjects. The changes in vergence angle (0.1°-0.2°) seem compatible with the tolerance range is Panum’s fusion area (15-30 arcmin). The absence of modulation in eye vergence during the control task signifies that it does not reflect visual stimulation but argues for a perceptual origin of the vergence modulation in the experimental task. That is to say, to change the angle of eye vergence visual stimulation alone is not sufficient. It necessitates attention of the subject.

A possible explanation for our results is to consider the distance of the peripheral target location, which is slightly further away from the eyes than the central fixation point. After the presentation of the cue, subjects focus on the peripheral target to fuse both retinal images of the target while maintaining fixation at the central point. According to such idea, the eyes diverge to a more distant plane and the angle of eye vergence decrease after cue/stimulus presentation. However, we found an increase in vergence angle, which argues against a near-triad explanation. This conclusion is supported by our previous paper [9] were we placed targets at different eccentricities (7° and 14° from the fixation point). The results of this study showed that for all targets the strength of the modulation in vergence was similar.

Recently we proposed a new role of eye vergence movements in attention and found that modulation in eye vergence relates to shifts in visuospatial attention [9]. Vergence modulation may follow the shift in attention [22] or may just co-vary with or even cause orienting attention. For instance, in our previous paper we provided evidence that eye vergence starts to modulate around the same time as subjects start to shift their attention [9]. Thus our current findings may be explained in terms of attentional differences between FI and FD subjects. The idea that perceptual style is ascribed to distinctive attentional abilities [15-17] agrees with such notion. Some studies suggest that FD subjects have greater difficulty in maintaining attention on a given aspect of information and in attending selectively to relevant cues, particularly in the presence of distracting elements [16,23]. Moreover, depending on the perceptual style subjects seem to attend to different aspects of information: FD subjects tend to focus their attention on global aspects of the information to be processed, while FI subjects tend to focus on partial aspects, e.g. [24,25]. Also, FD subjects are less effective in using their attentional resources resulting in poorer performance compared FI observers [26-28]. In contrast, other studies found no difference in stimulus detection performance, which indicates similar attention ability for FI and FD observers [29].

The zoom lens model [19,20] predicts an adjustment of the attention focus depending on the demanding task. So, this theory suggests a trade-off between the size of covered region by attention and processing efficiency of sensory processing because of limited processing capacities. Müller et al [30] found results according to this physiological prediction: while the extent of activated retinotopic visual cortex increased with the size of the attended region, the level of neural activity in a given sub-region decreased. Therefore a possible albeit a speculative explanation for the difference in eye vergence modulation between FI and FD observers is the different extent of activated visual area. The stronger increase in eye vergence modulation in the FI group could reflect a smaller size of the attended region. This smaller size of the attended region could explain the better performance behaviour of this group, which agrees with the notion that FI subjects are not so influenced by the context of a stimulus and are more biased towards local stimuli.

An alternative explanation for the faster reaction times observed in FI subjects may relate to the velocity or efficiency of stimulus processing [31]. The detection performance of FI and FD subjects in the first task was similar. Thus, we assume that the detection performance of the peripheral target in the second task was also similar for both groups. Therefore, the observed difference in reaction times to the central stimulus between the FI and the FD group in the second task is likely an outcome of dissimilar memory capacity, decision-making, and/or speed of stimulus processing. Accordingly, this means that the observed difference in vergence modulation between the FI and the FD subjects before the presentation of the central target relates to a difference in velocity or efficiency of stimulus processing and not to a difference in orienting attention. This idea may also explain vergence modulation in the first task. The cue stimulus induces the eyes to convergence thereby preparing the visual system for upcoming sensory information. However, the speculated link between vergence modulation and enhancement of stimulus processing needs to become spatial specific, i.e. to a single target. Otherwise in the no-cue condition vergence modulation is expected to occur as well. Thus, the improved reaction times observed in FI subjects may be explained by a superior preparatory phase. We speculate that the modulation in vergence eye movements before target onset could have a role in the switch in cortical state that has been observed in the visual cortex to prepare the visual system for new incoming sensory information leading to rapid or more efficient stimulus processing [21].

In conclusion, it becomes clear that small fixational eye movements have various roles in visual perception. In this regard, eye vergence movements, besides depth perception, may have a role in visual attention. However, further studies should elucidate this relationship.

## Materials and Methods

### Ethics Statement

The study was approved by the Ethics committee of the Faculty of Psychology of the University of Barcelona in accordance with the ethical standards laid down in the 1954 Declaration of Helsinki. All observers gave written informed consent before participating.

### Participants

To test vergence in relation to perceptual differences we selected eight FD and FI subjects (mean age: 21.88; STD: 3.48) at the extremes of the distribution of the scores of 157 participants tested with the 3rd section of GEFT [32]. High scores on this test are indicative for a local processing style, while low scores indicate a bias for global processing. As a criterion for extremity we used a percentage of correct responses (hits) to select the ~5% best or worst subjects of the population. To belong to the FD group, performance must be less than 50%, and to belong to the FI group performance should exceed 80%. All the participants were naïve to the purpose of the study and all had normal or corrected-to-normal vision. Participants received credit points or money for taking part in the experiment.

### Apparatus

The stimuli were displayed on a PC Pentium-IV 3000 with a Phillips Brilliance 22″ (CRT) screen. The display resolution was 24 pixels per degree (size: 1024 x 768 pixels or 27.6° x 20.7°). We used in-house C++ software for presenting the stimuli. The participants’ position of gaze was monitored using a binocular EyeLink II eye-tracking system at 500 Hz (SR Research System, Ontario, Canada). To compensate for any head movements, we used individually molded bite bars (UHCOTECH Head Spot, University of Houston, Texas, USA).

### Stimuli and procedure

Stimuli consisted of a fixation cross (5x5 pixels) surrounded by 8 peripheral bars (3x11 pixels), with an eccentricity of 7.5°. The stimuli were black on a grey background. Participants were sitting in a dimly lighted room, at 47 cm of the PC. Both experiments consisted of 4 sets of 32 trials for each task. Before starting the task, some training trials were conducted.

### Task 1

Observers were required to fixate to the central cross. After 300 ms after start fixation, 8 peripheral bars appeared. Then after 1000 ms, a cue (red line pointing to one of the peripheral bars, 3x13 pixels) or a no-cue (a red cross, 13x13 pixels) stimulus appeared for 100 ms at the central position (Figure 2a). The cue indicated (100% valid) the target. After an additional period of 1000 ms, one of the peripheral bars (target) briefly (100 ms) changed its orientation (tilt of 20° to the left or right). Participants had to respond by a button press as fast and accurately as possible whether the target tilted to the left or to the right. Feedback was not given to the observers.

### Task 2

Observers were required to fixate to the central cross. After 300 ms, 8 peripheral bars appeared, and after 1500 ms, one of the peripheral bars (peripheral target) changed briefly (50 ms) its orientation (20°). After additional fixation period of 1500 ms, a tilted bar (20°) appeared (for 50 ms) at the fixation cross position (central target). Participants responded with a button press (2 alternative choice) if they detected a match or a non-match in the orientation (Figure 2b). In an additional control experiment, the subjects viewed the same visual stimuli sequence. However, the subjects were instructed to fixate the central cross without performing any task.

### Eye data analysis

While subjects fixated the central cross, we calculated the angle of eye vergence as described in [9]. For the calculation of both eye gaze vectors we used the real distance from the screen to the observer and the actual inter-pupil distance. For each subject, the eye vergence data were normalized by dividing the raw data by the maximum value of the recorded samples from fixation onset to target onset. Only correct trials were analyzed. For the calculation of the mean eye vergence angle, we selected per subject a window of 100 ms around the maximum peak modulation in the average vergence angle, i.e. 550 to 650 ms after the onset of the cue/no-cue stimulus (Task 1) or change of the peripheral stimulus (Task 2).
